# A New Intelligent Medical Decision Support System Based on Enhanced Hierarchical Clustering and Random Decision Forest for the Classification of Alcoholic Liver Damage, Primary Hepatoma, Liver Cirrhosis, and Cholelithiasis

**DOI:** 10.1155/2018/1469043

**Published:** 2018-02-01

**Authors:** Aman Singh, Babita Pandey

**Affiliations:** ^1^Lovely Professional University, Department of Computer Science and Engineering, Jalandhar, Punjab 144411, India; ^2^Lovely Professional University, Department of Computer Applications, Jalandhar, Punjab 144411, India

## Abstract

Diagnosis of liver disease principally depends on physician's subjective knowledge. Automatic prediction of the disease is a critical real-world medical problem. This work presents an EHC-ERF-based intelligence-integrated model purposive to predict different types of liver disease including alcoholic liver damage, primary hepatoma, liver cirrhosis, and cholelithiasis. These diseases cause many clinical complications, and their accurate assessment is the only way for providing efficient treatment facilities to patients. EHC is deployed to divide the data into a hierarchy structure that is more informative for the disease predictions carried out by ERF. The occurrence of ERF error rate was dependent on correlation and strength of each individual tree where correlation is directly proportional to forest error rate and strength is inversely proportional to the forest rate. In total, two individual and three integrated classification models are developed to achieve enhanced predictions for the liver disease types. Analysis of results showed that the proposed framework achieved better outcomes in terms of accuracy, true positive rate, precision, *F*-measure, kappa statistic, mean absolute error, and root mean squared error. Furthermore, it achieved the highest accuracy rates when compared with the state-of-the-art techniques. Results also indicated that the weighted distance function employed in EHC has improved the efficiency of proposed system and has shown the capability to be used by physicians for diagnostic advice.

## 1. Introduction

The use of automatic diagnostic systems in medicine is increasing gradually [1]. Effectiveness of these systems has improved the judgment of physicians in predicting the sickness. Similar is the case with liver disease, whose occurrence has increased significantly in recent years. Applicability of intelligent computing algorithms to liver disorders has taken an enormous interest. Artificial neural network, fuzzy logic, rule-based reasoning, case-based reasoning, Fisher discriminant analysis, artificial immune recognition system, and decision tree algorithms have been widely applied in evaluating liver damage [2–26]. Development of these techniques has reduced the liver death rates and increased survival years in many patients.

Liver is the largest internal organ in the human body. It performs various metabolic functions such as detoxifying harmful chemicals, producing proteins, metabolizing drugs, clotting blood, storing glucose, producing cholesterol, and clearing bilirubin. Damage to any of the aforesaid function leads to liver disease [4]. Early symptoms of the disease are abdominal pain, nausea, poor appetite, fatigue, energy trouncing, and weight loss. Once the disease progresses, symptoms become more severe like edema, jaundice, ascites, abnormal bleeding, easy bruising, redness on the palms of hands, and sometimes memory confusion [3, 13, 16]. Most common causes of the disease are alcohol abuse, hepatitis viruses, iron overloading, abnormal genes, and Epstein-Barr virus [16, 27]. Liver disease can be classified into more than hundred types out of which viral hepatitis, liver cancer, primary biliary cirrhosis, liver fibrosis, neonatal hepatitis, primary hepatoma, alcoholic liver damage, nonalcoholic liver disease, cholelithiasis, liver cirrhosis, hemochromatosis, primary sclerosing cholangitis, tyrosinemia, and Wilson disease are usually prevalent [28].

This study is working on the classification of hepatobiliary disorders which include alcoholic liver damage, liver cirrhosis, primary hepatoma, and cholelithiasis. Alcoholic liver damage is an injury caused by high alcohol consumption. It starts occurring after an edge measurement of liquor intake is expended [29, 30]. Liver cirrhosis is a condition where the damage is irreversible. People with cirrhosis may create jaundice, itching, and outrageous tiredness. It is a dynamic infection, growing gradually over numerous years until, in the long run, it stops liver capacity [31, 32]. Primary hepatoma is a perilous tumor made out of cells that look like hepatocytes. It is ordinarily attached with cirrhosis and is currently the third major reason for liver cancer worldwide. It is often analyzed later because of the absence of pathognomonic side effects [33, 34]. Cholelithiasis is one of the most widely recognized surgical tissue around the world. Typically, a delicate balance exists between levels of cholesterol, phospholipids, and bile acids. When this balance is upset, there is predisposition for the expansion of lithogenic bile and the subsequent development of cholesterol-sort gallstones [35, 36].

A lot of work has been done on liver disease predictions using intelligent computing techniques but a very few studies have been found on classification of hepatobiliary disorders [28]. For these disorders, neurolinear and neurorule extraction techniques are developed where piece-wise linear discriminant functions are generated by the former and symbolic classification rules by later. Feedforward neural network with single hidden layer is selected for training and cross-entropy error function for improving the convergence. In comparison, NeuroLinear rules are found to be more concise and accurate [37]. Fuzzy multilayer perceptron network is built where the combination of membership values is given as input to MLP in the set categorization as low, medium, and high. The fuzziness incorporated enhanced neural network weights through backpropagating the errors [38]. An ensemble of neural networks is created where the output of the first level networks is used to train the second level networks [39]. A fuzzy model based on enhanced supervised fuzzy clustering algorithm is presented where global k-means method is used to initialize the fuzzy model. This method overcomes the limitation of simple k-means, that is, unknown number of clusters and random generation of initial positions of clusters [40]. Directed acyclic graph is integrated with neural network models to increase the diagnostic efficiency. The models include multilayer perceptron, support vector machines, radial basis function network, and random and pseudoinverse [41]. The structure of neural network is established using Darwinian genetic inheritance-based evolutionary process. Genetic search is being carried out for each generation in deciding network structure. Backpropagation learning determined the learning parameters and connection weights [42]. It is observed from the literature that a very limited work has been done on diagnosis of hepatobiliary disorders using intelligent computing algorithms.

This work proposes an intelligent medical decision support system for the classification of hepatobiliary disorders including alcoholic liver damage, primary hepatoma, liver cirrhosis, and cholelithiasis. The system is built using integration of data clustering and classification performed by enhanced hierarchical clustering and random decision forest algorithms, respectively. In total, two individuals and three integrated classification models are developed to achieve enhanced predictions for the disorders which include random decision forest, improved random decision forest, hierarchical clustering with random decision forest, hierarchical clustering with improved random decision forest, and enhanced hierarchical clustering with improved random decision forest. Performance of all aforesaid models is compared in terms of accuracy, true positive rate, precision, *F*-measure, kappa statistic, mean absolute error, and root mean squared error. Simulation results show that enhanced hierarchical clustering with improved random decision forest based-intelligence-integrated approach achieved better prediction outcomes than other individual and integrated models. Furthermore, it obtained higher accuracy rates when compared with the state-of-the-art techniques.

The paper is organized as follows.  Section 2 describes the proposed methodology developed to classify alcoholic liver damage, primary hepatoma, liver cirrhosis, and cholelithiasis.  Section 3 details the dataset used, discusses the experimental results, and compares the prediction performance of proposed approach with other classification models developed in this work and mentioned in literature. Finally, Section 4 briefly concludes the paper.

## 2. Proposed Methodology

**Figure 1:**
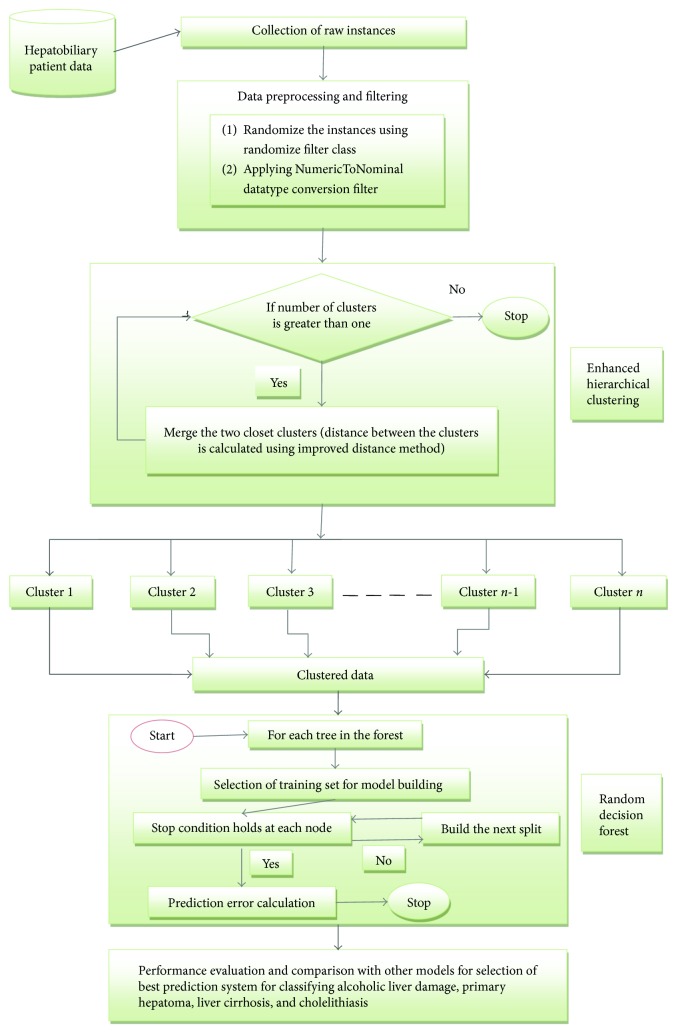
Block diagram of the proposed intelligence-integrated model for the classification of alcoholic liver damage, primary hepatoma, liver cirrhosis, and cholelithiasis.

The section aims to develop an intelligent medical decision support system based on hierarchical clustering and random decision forest algorithms for the classification of alcoholic liver damage, primary hepatoma, liver cirrhosis, and cholelithiasis. The prediction models deployed in the research work are represented as RF, ERF, HC-RF, HC-ERF, and EHC-ERF. RF stands for random decision forest with classification and regression tree algorithm as the learning model. ERF indicates improved random forest algorithm with random decision tree as the learning model. HC signifies hierarchical clustering algorithm with Euclidean distance function, and EHC denotes enhanced hierarchical clustering which used weighted distance function. HC-RF and HC-ERF indicate integration of hierarchical clustering with RF and ERF, respectively. EHC-ERF symbolizes the integration of enhanced hierarchical clustering with ERF which is the best classification model among all. Figure 1 illustrates the block diagram of the proposed intelligence-integrated model. Firstly, the hepatobiliary disorder data is taken as input in form of raw instances. The data incorporates five hundred and thirty-six instances with nine attributes and four target classes which are randomized first and then the sample values are converted from numeric to nominal format for giving input to the system. Secondly, enhanced hierarchical clustering algorithm is deployed to cluster the data. Then, the improved random forest algorithm with random decision tree as the learning model is used to predict alcoholic liver damage, primary hepatoma, liver cirrhosis, and cholelithiasis. Advantages of deploying hierarchical clustering and random decision forest algorithms include small cluster generation for better prediction, efficient handling of input variables, internal unbiased estimate of generalization error, deduction of key variables in classification, resistance to overtraining, and no apriori information needed about number of clusters. Owing to enhanced hierarchical clustering, the random decision forest predicts hepatobiliary disorder cases efficiently. Description of intelligent clustering and classification algorithms used in the proposed diagnostic model EHC-ERF is as follows.

Hierarchical clustering algorithm represents information by grouping data objects into hierarchy. Its structure is more instructive than the unstructured set of clusters returned by flat clustering. There is no apriori information needed about number of clusters required. It develops a sequence of nested clusters, and the range is from individual clusters of single points to all-together cluster [43–45]. This sequence of nested clusters is graphically represented by dendrogram where objects are grouped together step-by-step. For the hepatobiliary disorders data, a set of *M* data objects are given and *M* × *M* similarity matrix is calculated. Each item is assigned to a cluster. For *M* number of items, *M* clusters are formed. It finds the nearest cluster and joins them into a new single cluster. This decreases one cluster each time. Then, it calculates similarities between new cluster and each of old clusters. This process is repeated until there is only single cluster of size *M* × *M* is left. Before performing any clustering, it determines the proximity matrix which contains distance between each point using distance function. The procedure is described in Table 1 and Algorithm 1.

Where Euclidean function given in (1) is used to compute the distance in hierarchal clustering and a weighted distance function given in (2) is used in enhanced hierarchical clustering. For instance, the Euclidean distance (*d*) between vectors *p* = *p*_1_, *p*_2_,…, *p*_*n*_ and *q* = *q*_1_, *q*_2_,…, *q*_*n*_ in *n* space is represented as

(1) $$\begin{array}{c} d=\sqrt{\overset{n}{\underset{j=1}{\sum }}{\left(p_{j}-q_{j}\right)}^{2}}, \end{array}$$

and the improved distance between vectors *p* = *p*_1_, *p*_2_,…, *p*_*n*_ and *q* = *q*_1_, *q*_2_,…, *q*_*n*_ in *n* space is as follows:

(2) $$\begin{array}{c} d=\sqrt{{\left(v\right)}^{-1}\overset{n}{\underset{j=1}{\sum }}{\left(p_{j}-q_{j}\right)}^{2}} \end{array}$$

where *v* denotes weight, $\bar{p}$ indicates mean of attributes, and *v* is computed using the formula $v=\sum_{j=1}^{n}{\left(p_{j}-\bar{p}\right)}^{2}/n-1.$

Random forest algorithm constructs number of decision trees at training time and returns the output of class based on prediction of individual trees. The basic principle behind the classifier is forming a strong learner by a group of weak learners. It has the capability to create efficient classifiers by generating right kind of randomness. It resolves the problem of high bias and variance by finding average between two extremes [46–48]. Random forest formed with random input selection is called forest-RI. Occurrence of forest error rate is dependent on two factors: first is correlation and second is strength of each individual tree. Correlation is directly proportional to forest error rate, and strength is inversely proportional to forest rate. A tree acts as a strong classifier where error rate is low. Each tree is grown as per the following steps. In step 1, take *M* and *N* which represent number of training cases and number of variables, respectively. Step 2 finds a decision at node of tree, *n* of input variables are used where *n* < *N*. In step 3, training set for tree is picked *m* times with substitution from *M* training cases that are accessible. By predicting their classes, left cases are utilized to estimate the error of tree. In step 4, *n* factors are arbitrarily picked for every node of tree on which to make the choice at that node. On the basis of *n* variables presented in training data, calculate the finest split. Finally in step 5, each tree is grown to the maximum extent and there is no pruning. For predicting a new instance, the tree is traversed from top to bottom and then assigned a label associated with the training terminal node. This process is iterated over all trees, and the random forest classifier is obtained with majority vote among these classification trees. For instance, the hepatobiliary training data is represented as *D*_*m*_ = (*Y*_1_, *Z*_1_),…, (*Y*_*m*_, *Z*_*m*_) where *Y* and *Z* are independent random variables which are the same as the autonomous sample pair (*Y*, *Z*). This training set *D*_*m*_ is used to give estimation of *f*_*m*_ : [0, 1]^*k*^ → *R* of function *f*. Mean square error *f*_*m*_ is consistent if *H*[*f*_*m*_(*Y*) − *f*(*Y*)]^2^ → 0 as *m* → ∞. Input random vector *y* ∈ [0, 1]^*k*^, the aim is to predict response *Z* ∈ *R* by regression function approximation, that is, *f*(*y*) = *H*[Z | Y = y]. Random forest predictor consists of *F* randomized regression trees. The value predicted at query point *y* for *p*th tree in family is actually denoted by *f*_*m*_(*y*; *θ*_*p*_, *D*_*m*_) where *θ*_1_,…, *θ*_*f*_ are independent random variables. Before growing of individual trees, *θ* is used to resample the training data and to select the consecutive directions for partitioning. At this stage, different trees are combined to make finite forest estimate.

(3) $$\begin{array}{c} f_{F,m}\left(y;\theta_{1},\ldots ,\theta_{f},D_{m}\right)=\frac{1}{F}\overset{F}{\underset{p=1}{\sum }}f_{m}\left(y;\theta_{p},D_{m}\right). \end{array}$$

Since *F* may be chosen randomly high then let us assume *F* tends to infinity and the forest estimate is denoted as

(4) $$\begin{array}{c} f_{\infty ,m}\left(y;D_{m}\right)=H_{\theta }\left[f_{m}\left(y;\theta_{p},D_{m}\right)\right]. \end{array}$$

Here, *H*_*θ*_ denotes probability with respect to arbitrary factor *θ* which is conditional on *D*_*m*_. The process “*F* → ∞” is acceptable by large numbers and is conditional on *D*_*m*_.

(5) $$\begin{array}{c} \lim_{F\to \infty }f_{F,m}\left(y;\theta_{1},\ldots ,\theta_{f},D_{m}\right)=f_{\infty ,m}\left(y;D_{m}\right). \end{array}$$

In classification, response variable *Z* takes value in range [0, 1] and the value of *Z* is calculated with known variable *Y*. Classifier *f*_*m*_ is a measureable function of *y* and *D*_*m*_ and the label of *Z* is also approximated from *y* and *D*_*m*_. Classification and regression tree is used as the learning model in random forest algorithm, and random decision tree is used as the learning model in improved random decision forest. The classifier *f*_*m*_ is said to be consistent if conditional possibility of error *E*(*f*_*m*_) = *K*[*f*_*m*_(*Y*) ≠ *Z* | *D*_*m*_] satisfies $\lim_{m\to \infty }HE\left(f_{m}\right)=E^{*}$ where *E* ^∗^ is an unknown error but optimal bayes classifier is

(6) $$\begin{array}{c} f^{*}\left(y\right)=\left\{\begin{array}{l} 1,\;if\;K\left[Z=1\left|Y=y\right.\right]>K\left[Z=0\left|Y=y\right.\right],\\ 0,\;otherwise. \end{array}\right. \end{array}$$

The random forest classifier is obtained with majority vote among classification trees, that is,

(7) $$\begin{array}{c} f_{F,m}\left(y;\theta_{1},\ldots ,\theta_{f},D_{m}\right)=\left\{\begin{array}{l} 1,\;if\;\frac{1}{F}\overset{F}{\underset{p=1}{\sum }}f_{m}\left(y;\theta_{p},D_{m}\right)>\frac{1}{2},\\ 0,\;otherwise. \end{array}\right. \end{array}$$

## 3. Results and Discussion

The hepatobiliary disorder dataset obtained from a university-affiliated hospital in Japan is used for experimentation. The dataset includes nine attributes (continuous real-valued measurements from biomedical test), four classes, and five hundred and thirty-six instances. Attributes contain information about glutamic oxaloacetic transaminase, glutamic pyruvic transaminase, lactate dehydrogenase, gamma-glutamyl transpeptidase, blood urea nitrogen, mean corpuscular volume of red blood cells, mean corpuscular hemoglobin, total bilirubin, and creatinine. Four target classes include alcoholic liver damage, primary hepatoma, liver cirrhosis, and cholelithiasis. Each instance in the data represents information of a single male or female. The dataset is randomly split into training set containing seventy percent of data and test set containing remaining thirty percent. This division validates the proposed diagnostic model and reduces the biasness associated with instances. Table 1 details the description of biomedical test attributes and their measurement unit.

Obtained results of the developed individual and integrated classification models are compared using accuracy, true positive rate, precision, *F*-measure, kappa statistic, mean absolute error, and root mean squared error. Principally, the output of a classification model is produced in the form of TP, TN, FP, and FN; and then, the aforesaid parameters are calculated using these values. TP indicates true positive (diseased people correctly recognized as diseased), TN is true negative (normal people correctly recognized as normal), FN is false negative (diseased people incorrectly identified as normal), and FP expresses false positive (normal people incorrectly identified as diseased). Accuracy is the ability to distinguish target classes correctly. It is calculated using the ratio of sum of all TP and TN to sum of all TP, TN, FP, and FN. True positive rate is also known as sensitivity or recall which measures the proportion of instances that are correctly classified as class A, among all truly class A instances. It is computed using the ratio of TP to sum of TP and FN. Precision is also known as positive predictive value which measures the proportion of instances that truly belong to class A, among all classified class A instances. It is calculated using ratio of TP to sum of TP and FP. *F*-measure is also known as *F*-score which computes performance of a model for positive class. It is calculated using the ratio of multiplication of both precision and recall with 2 to sum of precision and recall. Kappa statistic computes the agreement of prediction with true class. Agreement is scaled between 0.0 and 1.0 where the later value signifies complete agreement. Mean absolute error is an average of absolute errors which is not squared before averaging and it is used to quantify the closeness of predictions to the eventual outcomes. Unlike MAE, root mean squared error squares the difference between predictions and eventual outcomes before averaging absolute errors in order to assign more weight to large errors.

**Figure 2:**
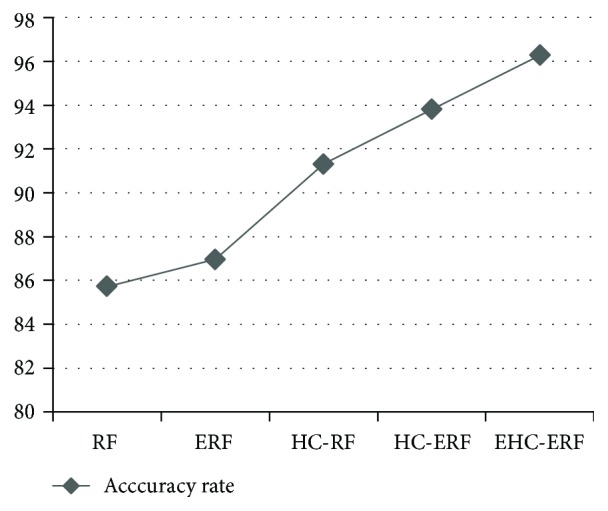
Comparative view of accuracy rates.

**Figure 3:**
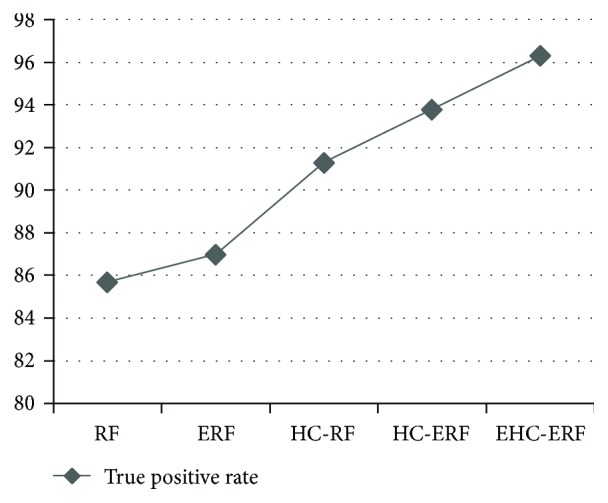
Comparative view of true positive rates.

**Figure 4:**
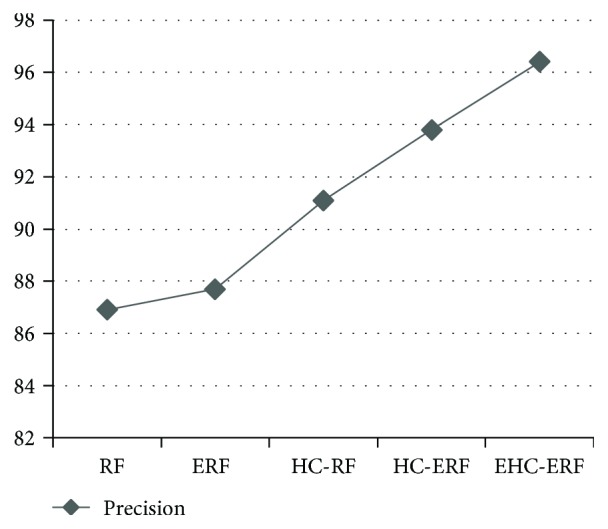
Comparative view of precision rates.

**Figure 5:**
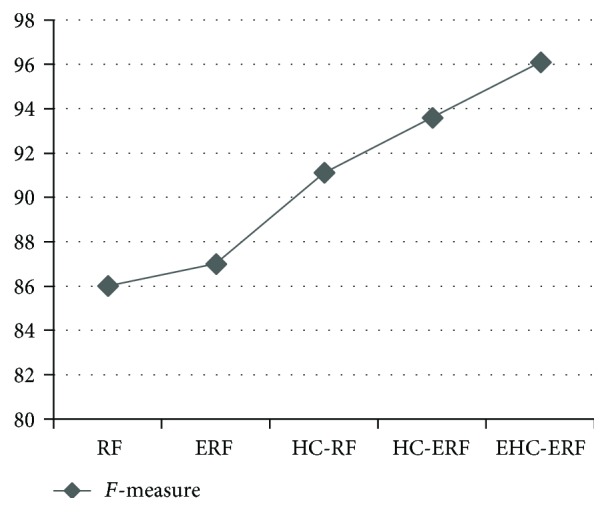
Comparative view of *F*-measure rates.

**Figure 6:**
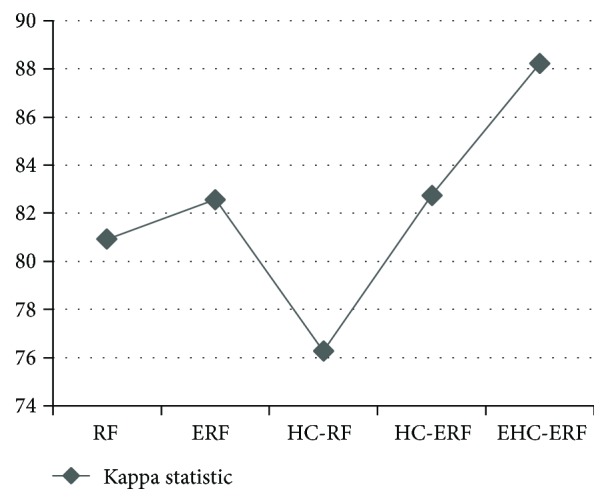
Comparative view of kappa statistic.

**Figure 7:**
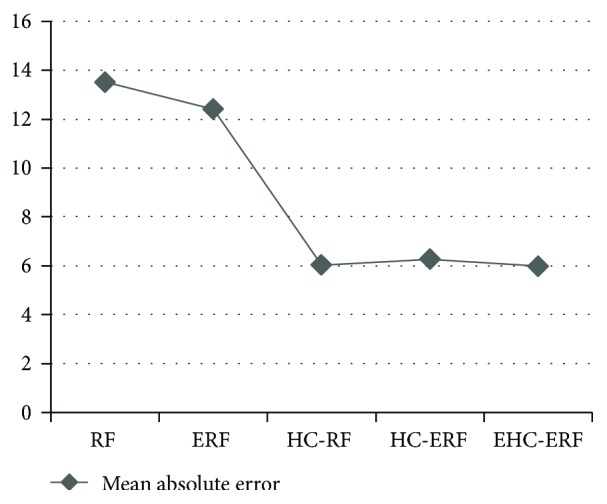
Comparative view of mean absolute error.

**Figure 8:**
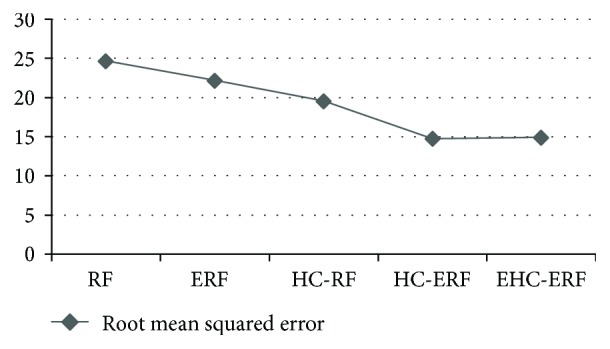
Comparative view of root mean squared error.

The intelligent diagnostic approaches built for predicting hepatobiliary disorders are represented as RF, ERF, HC-RF, HC-ERF, and EHC-ERF. RF stands for random forest algorithm, ERF signifies improved random forest algorithm, HC-RF indicates integration of hierarchical clustering with RF, HC-ERF stands for integration of hierarchical clustering with ERF, and EHC-ERF symbolizes the integration of enhanced hierarchical clustering with ERF. Figures 2, 3, 4, 5, 6, 7, and 8 illustrate the performance comparison among build classification models using accuracy, true positive rate, precision, *F*-measure, kappa statistic, mean absolute error, and root mean squared error rates, respectively.

Figure 2 depicts that RF had 85.71% accuracy, ERF had 86.96% accuracy, HC-RF had 91.3% accuracy, HC-ERF had 93.79% accuracy, and EHC-ERF had 96.27% accuracy.  Figure 3 shows that RF had 85.7% true positive rate, ERF had 87% true positive rate, HC-RF had 91.3% true positive rate, HC-ERF had 93.8% true positive rate, and EHC-ERF had 96.3% true positive rate.  Figure 4 portrays that RF had 86.9% precision, ERF had 87.7% precision, HC-RF had 91.1% precision, HC-ERF had 93.8% precision, and EHC-ERF had 96.4% precision.  Figure 5 describes that RF had 86% *F*-measure, ERF had 87% *F*-measure, HC-RF had 91.1% *F*-measure, HC-ERF had 93.6% *F*-measure, and EHC-ERF had 96.1% *F*-measure.  Figure 6 represents that RF had 80.92% kappa statistic, ERF had 82.57% kappa statistic, HC-RF had 76.27% kappa statistic, HC-ERF had 82.75% kappa statistic, and EHC-ERF had 88.23% kappa statistic.  Figure 7 depicts that RF had 13.52% mean absolute error, ERF had 12.41% mean absolute error, HC-RF had 6.04% mean absolute error, HC-ERF had 6.27% mean absolute error, and EHC-ERF had 5.99% mean absolute error.  Figure 8 presents that RF had 24.68% root mean squared error, ERF had 22.17% root mean squared error, HC-RF had 19.56% root mean squared error, HC-ERF had 14.74% root mean squared error, and EHC-ERF had 14.9% root mean squared error.

To select the most efficient medical decision support system for the classification of alcoholic liver damage, primary hepatoma, liver cirrhosis, and cholelithiasis; results of all developed models are compared (Table 2). It is observed that RF- and ERF-based models have not shown significant prediction performance. Although HC-RF and HC-ERF attained enhanced accuracy rates than the aforesaid models, EHC-ERF achieved the highest among all and is selected as the best classification model. Prediction results of EHC-ERF are also compared to other hepatobiliary classification methods mentioned in the literature. Hayashi et al. [37] stated that LDA, fuzzy neural network, NeuroRule, and NeuroLinear achieved accuracy rates of 63.2%, 77.3%, 88.3%, and 90.2%, respectively. In FNN, the backpropagation neural network model is applied where the input data is in the form of fuzzy arithmetic and fuzzy numbers. Pal and Mitra [38] mentioned that fuzzy multilayer perceptron network attained 76.0% and 88.9% accuracies for the best and second best choice criteria where the combination of membership values is given as input to MLP in the set categorization as low, medium, and high. The fuzziness incorporated enhanced neural network weights through backpropagating the errors. Hayashi and Setiono [39] mentioned that average accuracy rates of 30, 5, 10, and 15 neural networks are 90.27%, 90.92%, 91.78%, and 91.92%; average accuracy rates of developed biased neural networks are 92.64%, 92.02%, 93.25%, and 94.48%; average accuracy rates of applying neural networks as the second level model are 87.73%, 90.18%, 84.66%, 87.12%, 91.41%, 88.34%, and 89.57%. Ming et al. [40] presented a fuzzy model based on enhanced supervised fuzzy clustering algorithm where global k-means method is used to initialize the fuzzy model. This method overcomes the limitation of simple k-means, that is, unknown number of clusters and random generation of initial positions of clusters. Supervised fuzzy clustering with random initialization had 58.57% accuracy and enhanced supervised fuzzy clustering with global k-means had 58.78% accuracy. The proposed system also outperforms methods developed in the literature. The intelligence-integrated approach combines advantages of hierarchical clustering and random decision forest such as enhanced prediction results through generation of smaller clusters, consistency of cluster results on different algorithms runs, precise learning, estimation of key variables, fine computation of proximities between pairs of cases, and no apriori information required about cluster numbers.

## 4. Conclusions

Diagnosing a disease is one of the most difficult responsibility a clinician does have as one minute error can endanger patient life. Implementation of intelligent techniques has done a major transformation in predicting health examination data, and the medical domain has also been widely affected by this renovation. Classification of alcoholic liver damage, primary hepatoma, liver cirrhosis, and cholelithiasis disease is also an intricate task. As a part of constant efforts for making hepatobiliary disorder classification process well-organized and proficient, this research work developed an intelligence-integrated model based on enhanced hierarchical clustering and random decision forest algorithms. The model has advantages of both hierarchical clustering and random decision forest such as enhanced prediction results through generation of smaller clusters, consistency of cluster results on different algorithms runs, precise learning, estimation of key variables, fine computation of proximities between pairs of cases, and no apriori information required about cluster numbers. The integrated approach showed capability of improving complex medical decisions through clustered data. The prediction was carried out using a data of five hundred and thirty-six cases of hepatobiliary disorder. Simulation results confirmed the superiority of the proposed approach to other diagnostic models implemented in the study and mentioned in literature as well. Mean absolute error and root mean squared error rates were also small. Thousands people lose their lives because of erroneous evaluation and inappropriate treatment of alcoholic liver damage, primary hepatoma, liver cirrhosis, and cholelithiasis as the medical cases are still largely influenced by subjectivity of physicians. The proposed medical decision support system can be applied as a liver specialist assistant or as a model to train novice medical students. The system will also help physicians in evaluating complex cases that are otherwise hard to perceive. It has also shown the capability to reduce the need of liver biopsy to a possible extent.

## Figures and Tables

**Algorithm 1:**
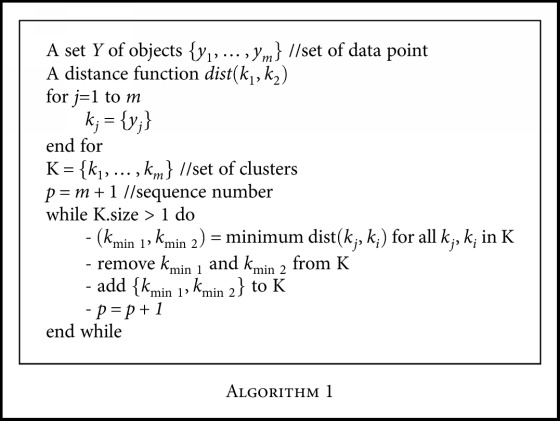
Algorithm 1

**Table 1:** The attributes of hepatobiliary disorder dataset.

Attribute	Description	Unit measurement
GOT	Glutamic oxaloacetic transaminase	Karmen unit
GPT	Glutamic pyruvic transaminase	Karmen unit
LDH	Lactate dehydrogenase	iu/l
GGT	Gamma glutamyl transpeptidase	*μ*/ml
BUN	Blood urea nitrogen	mg/dl
MCV	Mean corpuscular volume of red blood cells	fl
MCH	Mean corpuscular hemoglobin	pg
TBIL	Total bilirubin	mg/dl
CRTNN	Creatinine	mg/dl

**Table 2:** The simulation results of intelligence-integrated models.

Classification model	RF	ERF	HC-RF	HC-ERF	EHC-ERF
Accuracy	85.71%	86.96%	91.3%	93.79%	96.27%
TPR	85.7%	87%	91.3%	93.8%	96.3%
Precision	86.9%	87.7%	91.1%	93.8%	96.4%
*F*-measure	86%	87%	91.1%	93.6%	96.1%
Kappa statistic	80.92%	82.57%	76.27%	82.75%	88.23%
MAE	13.52%	12.41%	6.04%	6.27%	5.99%
RMSE	24.68%	22.17%	19.56%	14.74%	14.9%
